# Correction: Counterfactual thinking in psychiatric and neurological diseases: A scoping review

**DOI:** 10.1371/journal.pone.0307863

**Published:** 2024-07-23

**Authors:** Sofia Tagini, Federica Solca, Silvia Torre, Agostino Brugnera, Andrea Ciammola, Ketti Mazzocco, Roberta Ferrucci, Vincenzo Silani, Gabriella Pravettoni, Barbara Poletti

There are errors in the author affiliations. The correct affiliations are as follows:

Sofia Tagini^1^, Federica Solca^2^, Silvia Torre^1^, Agostino Brugnera^3^, Andrea Ciammola^1^, Ketti Mazzocco^4,5^, Roberta Ferrucci^6,7,8^, Vincenzo Silani^1,2^, Gabriella Pravettoni^4,5^, Barbara Poletti^1^

**1** Department of Neurology and Laboratory of Neuroscience, Istituto Auxologico Italiano IRCCS, Milan, Italy, **2** Department of Pathophysiology and Transplantation, “Dino Ferrari” Center, Università degli Studi di Milano, Milan, Italy, **3** Department of Human and Social Sciences, University of Bergamo, Bergamo, Italy, **4** Department of Oncology and Hemato-Oncology, University of Milan, Milan, Italy, **5** Applied Research Division for Cognitive and Psychological Science, IEO, European Institute of Oncology IRCCS, Milan, Italy, **6** Department of Health Sciences, Aldo Ravelli Center for Neurotechnology and Experimental Brain Therapeutics, International Medical School, University of Milan, Milan, Italy, **7** ASST Santi Paolo e Carlo, Neurology Clinic III, Milan, Italy, **8** Foundation IRCCS Ca’ Granda Maggiore Policlinico Hospital, Milan, Italy.
