# Supplementary material for: Counterfactual thinking in psychiatric and neurological diseases: A scoping review
Source: PLoS One. 2021 Feb 16;16(2):e0246388. doi: 10.1371/journal.pone.0246388 (PMC7886174; doi:10.1371/journal.pone.0246388)
Supplement: S1 Appendix — (DOCX) [file pone.0246388.s001.docx]

# **S1 Appendix.**

*PsycInfo:* (counterfactual AND (thinking OR thought OR reasoning) AND (disease OR illness OR (clinical AND (population OR sample)) OR impairment OR deficit OR patient*)).ti,ab.

*PubMed*: (counterfactual [Title/Abstract] AND (thinking [Title/Abstract] OR thought [Title/Abstract] OR reasoning [Title/Abstract]) AND (disease [Title/Abstract] OR illness [Title/Abstract] OR (clinical [Title/Abstract] AND (population [Title/Abstract] OR sample)) OR impairment [Title/Abstract] OR deficit [Title/Abstract] OR patient));

*Scopus*: TITLE- ABS - KEY (counterfactual AND (thinking OR thought OR reasoning) AND (disease OR illness OR (clinical AND (population OR sample)) OR impairment OR deficit OR patient));

*Web of Science*: (TS= (counterfactual AND (thinking OR thought OR reasoning) AND (disease OR illness OR (clinical AND (population OR sample)) OR impairment OR deficit OR patient*))).
